# Protein Data Bank: the single global archive for 3D macromolecular structure data

**DOI:** 10.1093/nar/gky949

**Published:** 2018-10-24

**Authors:** wwPDB consortium, Stephen K Burley, Helen M Berman, Charmi Bhikadiya, Chunxiao Bi, Li Chen, Luigi Di Costanzo, Cole Christie, Jose M Duarte, Shuchismita Dutta, Zukang Feng, Sutapa Ghosh, David S Goodsell, Rachel Kramer Green, Vladimir Guranovic, Dmytro Guzenko, Brian P Hudson, Yuhe Liang, Robert Lowe, Ezra Peisach, Irina Periskova, Chris Randle, Alexander Rose, Monica Sekharan, Chenghua Shao, Yi-Ping Tao, Yana Valasatava, Maria Voigt, John Westbrook, Jasmine Young, Christine Zardecki, Marina Zhuravleva, Genji Kurisu, Haruki Nakamura, Yumiko Kengaku, Hasumi Cho, Junko Sato, Ju Yaen Kim, Yasuyo Ikegawa, Atsushi Nakagawa, Reiko Yamashita, Takahiro Kudou, Gert-Jan Bekker, Hirofumi Suzuki, Takeshi Iwata, Masashi Yokochi, Naohiro Kobayashi, Toshimichi Fujiwara, Sameer Velankar, Gerard J Kleywegt, Stephen Anyango, David R Armstrong, John M Berrisford, Matthew J Conroy, Jose M Dana, Mandar Deshpande, Paul Gane, Romana Gáborová, Deepti Gupta, Aleksandras Gutmanas, Jaroslav Koča, Lora Mak, Saqib Mir, Abhik Mukhopadhyay, Nurul Nadzirin, Sreenath Nair, Ardan Patwardhan, Typhaine Paysan-Lafosse, Lukas Pravda, Osman Salih, David Sehnal, Mihaly Varadi, Radka Vařeková, John L Markley, Jeffrey C Hoch, Pedro R Romero, Kumaran Baskaran, Dimitri Maziuk, Eldon L Ulrich, Jonathan R Wedell, Hongyang Yao, Miron Livny, Yannis E Ioannidis

## Abstract

The Protein Data Bank (PDB) is the single global archive of experimentally determined three-dimensional (3D) structure data of biological macromolecules. Since 2003, the PDB has been managed by the Worldwide Protein Data Bank (wwPDB; wwpdb.org), an international consortium that collaboratively oversees deposition, validation, biocuration, and open access dissemination of 3D macromolecular structure data. The PDB Core Archive houses 3D atomic coordinates of more than 144 000 structural models of proteins, DNA/RNA, and their complexes with metals and small molecules and related experimental data and metadata. Structure and experimental data/metadata are also stored in the PDB Core Archive using the readily extensible wwPDB PDBx/mmCIF master data format, which will continue to evolve as data/metadata from new experimental techniques and structure determination methods are incorporated by the wwPDB. Impacts of the recently developed universal wwPDB OneDep deposition/validation/biocuration system and various methods-specific wwPDB Validation Task Forces on improving the quality of structures and data housed in the PDB Core Archive are described together with current challenges and future plans.

## INTRODUCTION

The Protein Data Bank (PDB, pdb.org) was established in 1971 as the first open-access, molecular data resource in biology (1). More than 47 years later, the PDB continues to serve as the single global repository for atomic-level, 3D structure data, making >144 000 experimentally-determined structures of proteins, DNA, and RNA, and their complexes with metal ions, drugs, and other small molecules freely available without restrictions on use. Since 2003, the PDB has been managed jointly by the Worldwide Protein Data Bank (wwPDB) consortium (2), including the US Research Collaboratory for Structural Bioinformatics Protein Data Bank (RCSB PDB; rcsb.org) (3), the Protein Data Bank in Europe (PDBe; pdbe.org) (4), Protein Data Bank Japan (PDBj; pdbj.org) (5) and BioMagResBank (BMRB; www.bmrb.wisc.edu) (6). The wwPDB partners are committed to ensuring adherence to the **FAIR** Principles of **F**indability-**A**ccessibility-**I**nteroperability-**R**eusability (7).

Today, the PDB is universally regarded as a core data resource essential for understanding the functional roles that macromolecules play in biology and medicine. Publication of new macromolecular structures in most scientific journals is contingent on mandatory deposition to the PDB of the 3D atomic coordinates comprising the structural model plus experimental data used to derive the structures and associated metadata. Many governmental and non-governmental research funders also require PDB deposition of unpublished macromolecular structure data. All of these 3D structural data are stored in one of two wwPDB Core Archives. The PDB Core Archive houses 3D atomic coordinates of >144 000 structural models of proteins, DNA/RNA, and their complexes with metals and small molecules. The PDB Core Archive also houses related experimental data/metadata from Macromolecular Crystallography (MX). The BioMagResBank (BMRB; www.bmrb.wisc.edu) Core Archive houses related experimental data/metadata from Nuclear Magnetic Resonance spectroscopy (NMR). The wwPDB partners work closely with the Electron Microscopy Data Bank (EMDB; emdb-empiar.org), which houses related experimental data/metadata from 3D Electron Microscopy (3DEM) and Electron Tomography (ET).

**Figure 1.**
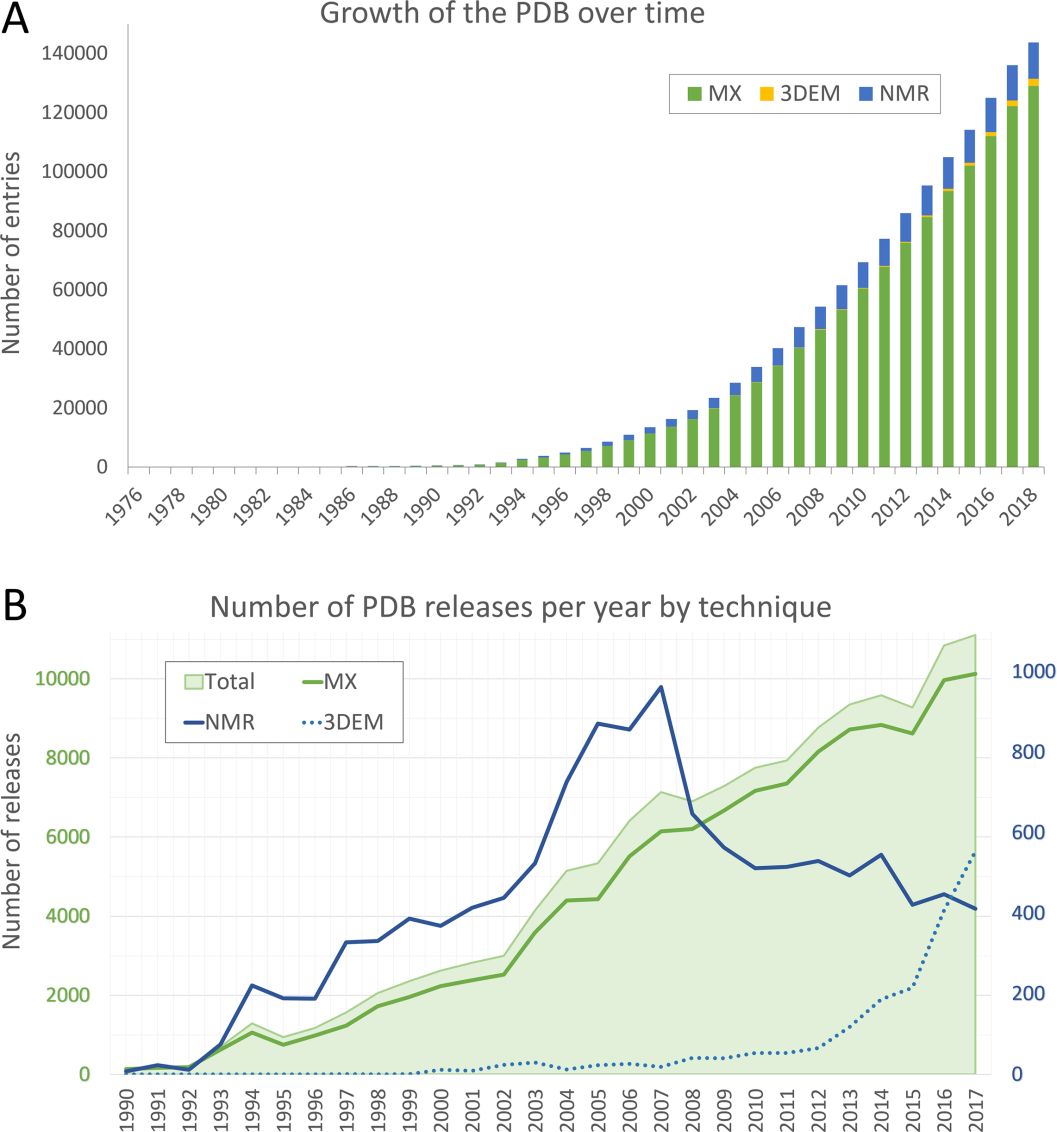
(**A**) Growth the PDB Core Archive. Total height of each bar indicates aggregate released structures, coloured by experimental technique (MX—green, 3DEM—yellow, NMR—blue). (**B**) Number of PDB structures released annually. All PDB Core Archive structures are indicated with light green shading, and MX structures are shown with a solid green line, plotted with respect to the green primary axis (left). NMR structures (blue solid line) and 3DEM structures (blue dashed line) are plotted with respect to the blue secondary axis (right).

The PDB Core Archive has seen steady growth since its inception, with over 11,000 new structures plus experimental data/metadata released in 2017 (Figure 1A). In aggregate, most of the 3D structures (89.5%) in the PDB Core Archive were determined using macromolecular crystallography (MX), with the remainder determined by NMR (8.5%), 3DEM (1.6%), and other techniques (0.4%). These overall metrics mask recent trends, which show that in 2016 3DEM overtook NMR as the second most popular technique for determining atomic level structures (Figure 1B).

While the PDB Core Archive has grown enormously in scale and scope over the past 47 years and its management has evolved concurrently, adherence to the principle of open access and commitment to community engagement (1) continue to this day.

The Vision of the wwPDB is to:

- Sustain freely accessible, interoperating Core Archives of structure data and metadata for biological macromolecules as an enduring public good to promote basic and applied research and education across the sciences.

The Mission of the wwPDB is to:

- Manage the wwPDB Core Archives as a public good according to the **FAIR** Principles.
- Provide expert deposition, validation, biocuration, and remediation services at no charge to Data Depositors worldwide.
- Ensure universal open access to public domain structural biology data with no limitations on usage.
- Develop and promote community-endorsed data standards for archiving and exchange of global structural biology data.

PDB data are being used by researchers, educators, specialist bioinformatics resources and other Users from every inhabited continent and every UN-recognized sovereign nation. Nearly two million daily structure data file downloads from wwPDB partner websites and the two Core Archives attest to the important role that the wwPDB plays within the biological data ecosystem.

Ongoing collaborative work among wwPDB partners helps to ensure completeness, consistency, and accuracy of data in the two Core Archives. The wwPDB has also worked to enable growth of the corpus of structure data to accommodate new experimental techniques. Herein, we describe impacts of the recently developed universal wwPDB OneDep deposition/validation/biocuration system and various methods-specific wwPDB Validation Task Forces on improving the quality of structures and data housed in the PDB Core Archive, together with current challenges and future plans.

## PDB CORE ARCHIVE CONTENT

Unlike the situation in 1971, multiple techniques are now available for determining 3D structures of biological macromolecules. PDB structure depositions are currently restricted to atomic-level structures that have been substantially determined by one or more of the following supported experimental techniques: MX, NMR, 3DEM, powder diffraction and fiber diffraction.

### Atomic coordinate data

Every PDB structure deposition includes the atomic coordinates defining the 3D structural model of the macromolecule. Atomic positions are specified as Cartesian coordinates (*x, y*, *z*) using Ångström units (i.e. 0.1 nm) and a right-handed coordinate system. Additional method-specific attributes are provided for individual atoms (e.g. *B*-factors or temperature-factors for MX structures).

### Related metadata

To ensure adherence to the **FAIR** Principles (7), the atomic coordinates of 3D structures must be adequately described with additional mandatory metadata. These metadata include a hierarchy of information describing whether a particular atom is part of a polymer (and if so, which residue), or a metal ion, a ligand, a small molecule solute or a water molecule. For macromolecules, additional metadata including name, source organisms, and cross-references to other bioinformatics resources are provided. Data on the type of structure determination experiment performed and on the nature and production of the experimental sample are also archived. Consistent collection of structure data and experimental data/metadata, governed by defined vocabularies, allows Users of the PDB Core Archive to find and understand 3D structures of interest.

### Experimental data

Deposition of experimental data/metadata together with atomic coordinate data is required for all incoming structures. For MX experiments, deposition of structure factors or unmerged intensities and related metadata is required. These data are stored in the PDB Core Archive. For NMR experiments, deposition of assigned chemical shifts and geometric restraints and related metadata is required. These data and additional experimental data are stored in the BMRB Core Archive. For 3DEM experiments, Coulomb potential maps and related metadata are required. These data are stored in the EMDB (8). Experimental data/metadata accompanying MX, NMR and 3DEM structures are processed using the universal OneDep system for deposition/validation/biocuration (9). For NMR and 3DEM methods wherein experimental data are often deposited in advance of 3D structure determination, cross-referencing with subsequently deposited atomic coordinates ensures interoperability across the PDB and BMRB Core Archives and the EMDB. The OneDep system also allows Data Depositors to provide additional links to other experimental data, housed in repositories such as SBGRID (sbgrid.org) (10), IIRMC (proteindiffraction.org) (11), SASBDB (www.sasbdb.org) (12) and EMPIAR (www.ebi.ac.uk/pdbe/emdb/empiar) (13).

Mandatory archiving of the experimental data fulfils two important functions. First, it enables PDB Users to reproduce 3D structure determinations and analyses therefrom. Second, it allows quantitative assessments of how well the atomic coordinates conform to the experimental data. The wwPDB partnership has made significant investment in validation of 3D atomic-level structures together with experimental data/metadata (9, 14–19).

### Chemical reference data

In addition to atomic coordinates and experimental data, the wwPDB provides key chemical reference data including the Chemical Component Dictionary (CCD) (20) and Biologically Interesting Molecule Reference Dictionary (BIRD) (21). The CCD provides a detailed chemical description of every unique chemical component represented within 3D atomic coordinates in the PDB Core Archive, including standard and modified residues, metal ions, small molecule ligands, solute molecules, and water molecules. Each chemical component definition includes descriptions of chemical properties, such as stereochemical assignments, chemical structure descriptors (SMILES and InChI), systematic chemical names, chemical formulae, and idealized atomic coordinates. Currently, the BIRD includes detailed descriptions of biologically interesting peptide-like antibiotic and enzyme inhibitor molecules present in the PDB Core Archive. These molecules may be composed of a mixture of polymer and non-polymer components or short polymeric entities, and require a description on the level of the whole molecule and on the level of constituent parts. In future, the BIRD resource could be extended to other kinds of oligomeric molecules, which may require analogous dual definitions.

## PDBx/mmCIF DATA FILE FORMAT

Providing consistent and accurate representation for all 3D structures in the PDB Core Archive allows these data to be easily searched and exploited by Users around the world. Significant advances in structure determination techniques over the last decade have resulted in an increase in the size and complexity of macromolecular structures studied by structural biologists. As a result, it is no longer possible to represent these large macromolecular machines with the legacy PDB file format, which is restricted to a maximum of 99 999 atoms and 62 single-character polymer chain identifiers. For a time, 3D structural models that exceeded these limits were split into multiple PDB entries, causing considerable inconvenience to Data Depositors and Users alike. To address this limitation, the wwPDB convened a working group to obtain community support for adoption of a common extensible PDBx/mmCIF data archiving framework (22–24) with the associated mmCIF format as the master file format for the PDB Core Archive. Large structures that were previously split across multiple PDB entries were merged into a single PDB entry using the PDBx/mmCIF format. All 3D structures in the PDB Core Archive are now stored and distributed in PDBx/mmCIF format.

Where possible, the wwPDB has continued to make structures in the PDB Core Archive available in legacy PDB format for the convenience of the User community. For the avoidance of doubt, the legacy PDB format was ‘frozen’ in 2012, and no longer conveys the broad range of rich metadata represented in the PDBx/mmCIF files. Most major structural biology data resources and software tools have embraced the PDBx/mmCIF format, and continued reliance on the legacy PDB file format is strongly discouraged by the wwPDB.

Experimental data are distributed using various file formats, reflecting minor differences in the evolution of data archiving practices within different structural biology communities. Structure factors from MX experiments are stored and distributed in the PDBx/mmCIF format. Chemical shifts from NMR experiments are stored and distributed in the BMRB Core Archive NMR-STAR format (25, 26). Coulomb potential maps from 3DEM experiments are stored and distributed in the CCP4 map format, whereas the associated metadata are stored and distributed in EMDB-XML format (27).

All the data categories and items represented using the PDBx/mmCIF format are unambiguously defined in the PDBx/mmCIF dictionary (mmcif.wwpdb.org) (22). These data definitions include relationships among different data categories, and allowed data types for all data items in the data categories. The PDBx/mmCIF dictionary also includes allowed enumerations and ranges of values for individual data items, thereby enabling validation of the data items in each PDBx/mmCIF file across the PDB Core Archive. Finally, the PDBx/mmCIF format has the advantage of being fully extensible, enabling archiving of new data types as structural biology continues to develop as a scientific discipline.

### Additional structure data file formats

In addition to PDBx/mmCIF, the wwPDB also distributes the atomic coordinates of every PDB structure in PDBML/XML format, which can be read using a standard XML parser (pdbml.pdb.org) (28) and as RDF (rdf.wwpdb.org/pdb) (5,29).

## GLOBAL DATA DEPOSITION

**Figure 2.**
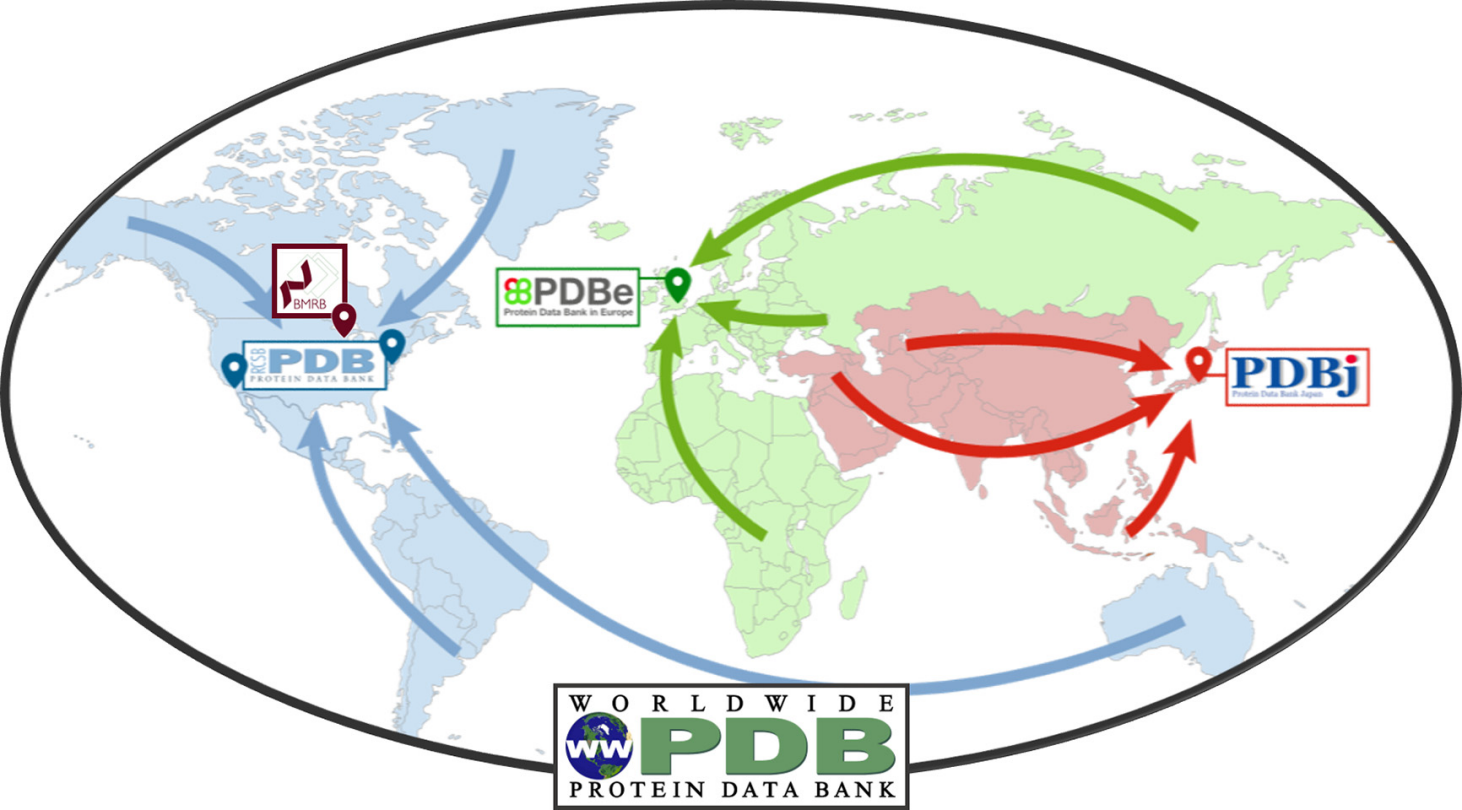
World map indicating the locations of wwPDB partner sites and color-coded to indicate the regions from which each accepts PDB depositions.

In 2014, the global OneDep deposition-validation-biocuration system was launched (9). This important advance ensured that all wwPDB regional data centers (RCSB PDB, PDBe and PDBj) provide the same data processing experience to Data Depositors around the world. The OneDep system processes depositions of 3D structures coming from all of the experimental methods currently supported by PDB Core Archives. OneDep also supports all EMDB depositions, including Coulomb potential maps that are not accompanied by 3D atomic coordinates. Validation and biocuration of depositions is geographically distributed, with RCSB PDB processing all depositions from the Americas and Oceania, PDBe processing depositions from Europe and Africa, and PDBj processing all depositions from Asia and the Middle East (Figure 2). This arrangement distributes validation/biocuration efforts across three wwPDB partner sites, and allows most Data Depositors to communicate with wwPDB biocurators located in the same or nearby time zones.

To improve data quality and to allow for future extensibility, the OneDep system uses the PDBx/mmCIF framework throughout deposition-validation-biocuration. The launch of the OneDep system was accompanied by a significant extension of the PDBx/mmCIF dictionary, including addition of new or updated enumerations and allowed ranges for individual data items and significant changes to improve representation of 3DEM structures within the PDB Core Archive. Both the PDBx/mmCIF dictionary and the OneDep system undergo continuous updates, with enumerations being extended and new data items being added when and where appropriate.

wwPDB validation (15) and biocuration (30) processes have been described in detail, and information about the deposition and biocuration policies and procedures is available on the wwPDB website (www.wwpdb.org/documentation/biocuration). Recently, validation of ligands in the PDB Core Archive was improved with the adoption of a more robust way of flagging those molecules that do not fit electron density well (31). The wwPDB validation report provides consistent quality assessment metrics across the entire PDB archive, enabling comparisons between different structures. This allows Users to rank PDB structures relevant for their needs based on validation criteria.

Validation is performed throughout the deposition process: a preliminary validation report is provided to Data Depositors at the time of deposition; an updated, confidential report is then provided to Data Depositors once the biocuration is concluded; and a final public report is released alongside the PDB entry. An increasing number of scientific journals now require wwPDB validation reports to be included at the time of manuscript submission to assist referees in assessment of the quality of the 3D structure data. The wwPDB strongly encourages all Data Depositors to provide their confidential wwPDB validation reports when submitting related manuscripts.

Release of PDB entries requires either a request from the Data Depositor, a notification that the related manuscript has been published in a scientific journal, or the expiration of the on-hold period (currently maximum one year from the date of deposition), whichever occurs first.

The RCSB PDB currently serves as the designated PDB Core Archive Keeper, coordinating a two-phase weekly update procedure for both new and revised entries. Each wwPDB regional data center finalizes entries for the next weekly release up until Thursday at noon local time. At this time, data marked for release are compiled and re-checked at RCSB PDB and then distributed to all wwPDB partner sites. Phase 1 of the weekly update occurs every Saturday at 03:00 UTC, when the wwPDB website (www.wwpdb.org/download/downloads) makes the following data available for each new entry: amino acid and nucleotide sequences for each distinct polymer and, where appropriate, the InChI string(s) for each distinct ligand, and the crystallization pH value(s). Phase 2 of the weekly update occurs every Wednesday at 00:00 UTC, when the wwPDB FTP system and the FTP sites at wwPDB partner sites are updated to include all new and modified PDB entries and obsolete entries are removed from the active archive. Release of PDB data in two phases is intended to assist the computational biology community in operating various prediction challenges, including CASP (32), CAPRI (33), CAMEO (34) and D3R (35).

## DATA DISSEMINATION

The wwPDB website (www.wwpdb.org) provides news announcements, describes how to access PDB data, and hosts PDBx/mmCIF data dictionary resources.

**Figure 3.**
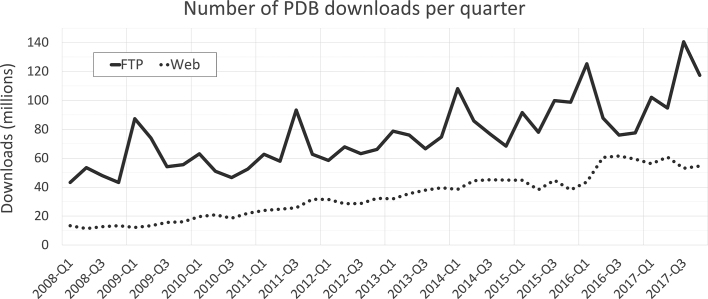
Quarterly wwPDB structure data file download metrics from 2008–2017 (FTP and rsync downloads-solid line; wwPDB partner website downloads-dotted line).

PDB data are made available from all wwPDB partner sites *via* FTP and also through their individual websites - RCSB PDB (rcsb.org), PDBe (pdbe.org), PDBj (pdbj.org), and BMRB (www.bmrb.wisc.edu). In 2017, the FTP archive recorded >450 million structure data file downloads, and at the individual wwPDB partner websites they numbered >220 million (Figure 3). The aggregate number of unique PDB Users (unique IP addresses) worldwide is conservatively estimated at >1 million.

Following consultation with PDB Users, the wwPDB implemented versioning of data in the PDB Core Archive. Automatic auditing of changes to PDB entries has been introduced, which distinguishes between updates to atomic coordinates, chemistry or polymer sequence (denoted as ‘major’) and other updates, including citation updates (denoted as ‘minor’). A versioned FTP archive (ftp://ftp-versioned.wwpdb.org) has been introduced, which serves up the latest minor version of each major version of a PDB entry. This provision allows Users access to information on updates to each PDB structure and allows for comparison of available major versions to review changes. In the versioned FTP, PDB entries are identified by an eight character ID allowing for extension of the PDB code beyond its current four characters.

All MX experimental data in the PDB Core Archive are distributed *via* the wwPDB FTP. Experimental data relating to 3D structures coming from NMR are also distributed *via* the wwPDB FTP. Additional NMR experimental data associated with 3D structures, not collected by OneDep but deposited at BMRB, are distributed by BMRB. The wwPDB FTP also mirrors the EMDB FTP, providing access to the entire contents of the EMDB.

## ARCHIVE UPDATES

To improve consistency of data within the PDB Core archive, wwPDB biocurators routinely update PDB entries with new or corrected metadata, such as citation information or updates triggered by changes to the CCD (30).

The wwPDB has undertaken several archive-wide remediations, starting with the standardization of the PDB Core archive in 2007. This major undertaking introduced chemical descriptors to the CCD and ensured that atom names of standard residues are consistent with IUPAC nomenclature (36). Subsequent remediations have focused on adding further metadata, including taxonomy information (2009), and on introducing missing molecular assembly information (2011). As noted above, in 2014, after the adoption of PDBx/mmCIF as the master format, structural models that spanned more than one PDB entry due to limitations of the historical PDB file format were combined into single PDB entries distributed exclusively in PDBx/mmCIF format.

Launch of the OneDep system has enabled, for the first time, large-scale remediation to improve data consistency across all entries in the PDB Core archive. Older PDB entries deposited using legacy deposition systems were updated to ensure that their metadata are consistent with newer entries deposited using the OneDep system (∼30% of older PDB entries underwent remediation). This effort also included better representation of data related to 3DEM entries. Remediation is an ongoing process, intended to ensure better data quality and consistency and improved searchability across the PDB Core Archives. Consistent representation of carbohydrates and post-translational modifications will be the focus of future remediation efforts.

## COMMUNITY ENGAGEMENT

The enduring value of the PDB Core archive to its large, global User community depends critically on data consistency, accuracy, and accessibility. It is, therefore, essential to ensure that the User needs are both understood and addressed. The wwPDB is guided by an expert international advisory board, which reviews the activities of the organization annually to ensure that the wwPDB is delivering value to its diverse User community. Outcomes of the annual advisory board meetings are published on the wwPDB website (www.wwpdb.org/about/advisory). This advisory board provides essential guidance for wwPDB developments and delivers community feedback on changes that can benefit the PDB archive.

The field of structural biology is constantly evolving, and the wwPDB is committed to staying abreast of these advances. Over the years, expert, method-specific wwPDB Validation Task Forces have been established for MX (19), NMR (17) and 3DEM (16). Each of these groups contributed to the development of the OneDep validation system and the wwPDB validation report. In collaboration with the Cambridge Crystallographic Data Centre (37) and the Drug Design Data Resource (D3R) (www.drugdesigndata.org), the wwPDB convened a Ligand Validation Workshop in 2015 to obtain recommendations regarding improved PDB ligand representation and validation (18). Further refinements of our validation processes are being guided by continued interaction with the validation task forces. The wwPDB validation report is updated annually to incorporate software updates, new validation processes, and to update the archive wide validation statistics by incorporating PDB entries from the previous year.

To establish data standards and obtain recommendations for improving data quality on rapidly developing experimental methods currently not supported within the PDB archive, the wwPDB has also established task forces for Small Angle Scattering (SAS) (38) and Integrative/Hybrid Methods (I/HM) (39), both of which have published their recommendations in white papers.

All of these efforts are underpinned by the PDBx/mmCIF Working Group (www.wwpdb.org/task/mmcif), which advises on data standards for representation of structural biology data in the PDBx/mmCIF dictionary. This working group meets regularly with representatives from the wwPDB to advise on adjustments to the data dictionary.

In consultation with the working group, the wwPDB has recently produced an extension to the PDBx/mmCIF dictionary to incorporate multiple crystal data collection techniques such as those used in serial femtosecond crystallography (SFX) and X-ray free electron laser (XFEL) experiments. This step increased the amount of metadata that will be available for future multiple crystal data collection experiments. None of this would have been possible had the organization not transitioned from the legacy PDB format to the PDBx/mmCIF data standard. The wwPDB will continue to expand and extend the PDBx/mmCIF dictionary as structural biology advances.

The wwPDB has also worked with the NMR community to develop the NMR Exchange Format (NEF) (40), and this format for deposition of NMR restraint data will be supported by the OneDep system in a later software release.

Communications from Data Depositors regarding their own depositions should only be submitted via the Communication Panel within the individual OneDep session. More general feedback regarding issues not directly related to an individual OneDep session is welcome at the wwPDB Customer Service Helpdesk—further contact information is available at deposit.wwpdb.org.

## CURRENT AND FUTURE CHALLENGES

**Figure 4.**
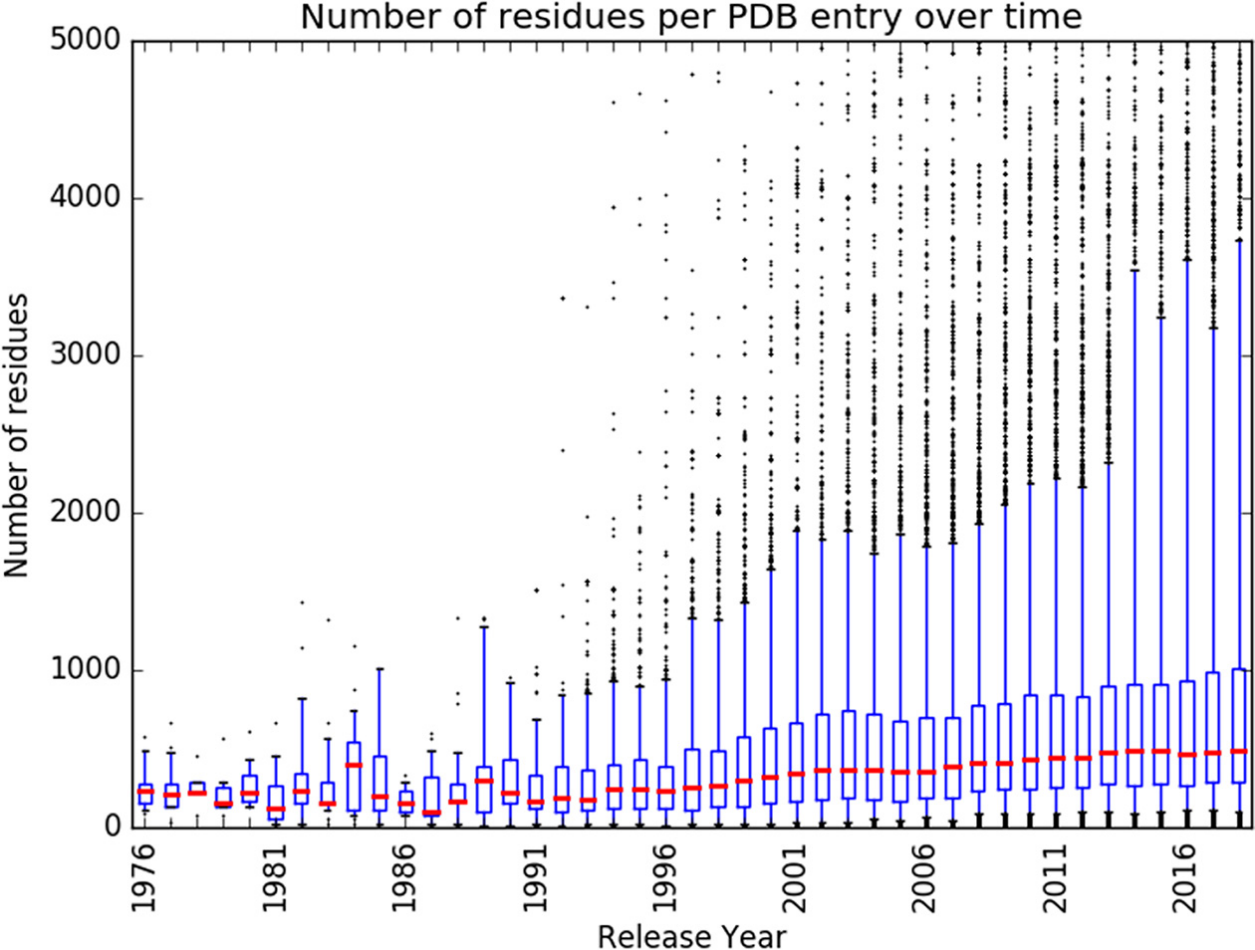
Boxplot representation of the number of residues per PDB structure per year. Red lines represent the median value, boxes represent the 25 and 75 percentile values, and whiskers represent the 5th and 95th percentile values.

The PDB Core Archive continues to grow year-on-year (Figure 1A), while incoming structures grow in size and complexity (Figure 4). The wwPDB partners have addressed these challenges by launching and continually improving the unified OneDep deposition–validation–biocuration system. Global adoption of best practices and increased automation have improved the Data Depositor experience and made wwPDB biocuration more efficient. Instead of devoting efforts to unnecessary repetitious work on relatively uncomplicated depositions, our biocurators are now able to focus more of their time on the more complex depositions, thereby improving data consistency and accuracy. ORCiD IDs (orcid.org) for OneDep Depositor(s) of Record were made mandatory in 2018. To further improve the depositor experience and enable better management of incoming data, OneDep protocols will be changed to allow login using ORCiD persistent digital identifier unique to each researcher.

Rapidly evolving experimental methods, such as SFX/XFEL and 3DEM, require frequent extensions of the PDBx/mmCIF data dictionary for expanded collection of related metadata using the OneDep system. Validation of 3DEM structural data also requires further development of OneDep, particularly for validation of 3D atomic coordinates against Coulomb potential maps that will allow better assessment of 3DEM structure quality. Emerging methods, such as Integrative/Hybrid Methods (I/HM), present entirely new challenges in data representation and validation. In 2014, the wwPDB established a wwPDB Hybrid Methods Task Force, which produced a white paper detailing the outcome of its inaugural meeting (39). These recommendations led to development of a prototype system (PDB-Dev, pdb-dev.wwpdb.org) for representation, deposition, and archiving of I/HM structure data (41, 42). In parallel, as a first step towards inclusion of I/HM structures in the PDB Core Archive, the OneDep system, in partnership with SASBDB (12), recently introduced combined data deposition for structures determined using SAS data in addition to the use of traditional structure determination techniques.

To avoid fragmentation of structural biology data in different archives that do not interoperate with one another, wwPDB partners are leading efforts to coordinate archiving activities across the discipline. Going beyond the two Core Archives, the PDBx/mmCIF data dictionary contains data items that provide links for individual 3D structures with related data stored by other specialist data resources, such as SBGRID (10), IIRMC (11) and EMPIAR (13).

## PLANS FORWARD

The wwPDB is committed to ensuring that all data in the PDB Core archive are as accurate and consistent as possible. Three major initiatives are planned for the coming years.

First, Depositors of Record will be able to make corrections to existing structures in the PDB Core Archive by updating the atomic coordinates, while preserving the original PDB identifier to improve ligand structures or make a better quality structure available for a particular macromolecule. The recent introduction of versioning makes this long-desired opportunity feasible for the first time.

Second, work is underway to further enhance the original wwPDB validation report. Of particular importance will be enhanced validation for both NMR and 3DEM structures. For NMR, the OneDep system will restrict deposition of restraints to the NEF (40) or NMR-STAR (25, 26) formats, and the future validation reports will include analysis of NMR experimental restraint data. The archival format for NMR restraint data will continue to be NMR-STAR. For 3DEM, the wwPDB partners are working with EMDB to improve validation of atomic models built using Coulomb potential maps. Finally, ligand representation and validation will be improved as recommended in the Ligand Validation Workshop white paper (31). All of this work will be informed by ongoing discussions with the various wwPDB Validation Task Forces and other community experts.

Third, the wwPDB plans to develop a new mechanism to resolve the official DOI for each PDB structure. Evolution of the wwPDB PDB Core Archives has resulted in there being multiple data files associated with a given 3D structure, including the atomic coordinates, experimental data, validation reports, and other associated files. The wwPDB partners plan to introduce a new wwPDB web page accessible from the official DOI that will provide access to all relevant files across the two Core Archives. The wwPDB strongly encourages all scientific journals to link to these pages using the DOI for each newly published 3D structure once these pages are made available.

## HISTORICAL PERSPECTIVE AND POSTSCRIPT

The PDB has undergone enormous changes since its humble beginnings with just seven structures in 1971. Notwithstanding seismic shifts in the discipline that we now call structural biology and 20,000-fold growth in the PDB, management of the resource as the single global archive of 3D structure data for biological macromolecules continues to be underpinned by an unwavering commitment to universal access to high data quality without limitations on usage. Initially, what is now referred to as the PDB Core Archive was managed entirely within the United States, first at Brookhaven National Laboratory (1), and then by the RCSB PDB, a consortium formed by Rutgers University, the San Diego Supercomputer Center/University of California San Diego and the National Institute of Standards and Technology (3). Since 1999, PDBj and PDBe (formerly known as Macromolecular Structure Database (MSD)) informally worked with RCSB PDB to support PDB deposition and biocuration. In 2003, this arrangement was formalized, and joint international management of the resource began with founding of the wwPDB by the RCSB PDB, PDBe, and PDBj (2). BMRB joined the wwPDB in 2006.

Above all since 2003, joint management by the wwPDB has ensured that the resource remains the single global archive for 3D macromolecular structure data, becoming a central player in the international biological data ecosystem. In addition, joint management has enabled a host of important accomplishments, including (i) adoption of the PDBx/mmCIF data dictionary, (ii) multiple rounds of archive-wide remediation to improve data consistency and data quality, (iii) mandatory deposition of experimental data, (iv) development of community standards for validation of structures and related data/metadata from multiple experimental methods, (v) launch of the universal OneDep deposition/validation/biocuration system and (vi) launch of the PDB-Dev prototype for archiving I/HM structures.

As in 2003, the wwPDB partners remain committed to working together with their diverse User communities to confront myriad challenges presented by ever more complex structures and related data/metadata. Many of the most exciting structures entering the PDB Core Archive today are determined using the rapidly evolving techniques of SFX/XFEL and 3DEM. The I/HM structures on the horizon promise to be even more important contributors to research and education in biomedicine as 3D structures of macromolecular machines at work inside cells come from cryo-electron tomography combined with other methods. Members of the wwPDB organization look forward to addressing these challenges and ensuring that joint management of the wwPDB Core Archives continues to serve Users around the globe.

## Current wwPDB Consortium Members with Affiliations

RCSB Protein Data Bank:

Stephen K. Burley^1,2,3^, Helen M. Berman^1^, Charmi Bhikadiya^1^, Chunxiao Bi^3^, Li Chen^1^, Luigi Di Costanzo^1^, Cole Christie^3^, Jose M. Duarte^3^, Shuchismita Dutta^1^, Zukang Feng^1^, Sutapa Ghosh^1^, David S. Goodsell^1,4^, Rachel Kramer Green^1^, Vladimir Guranovic^1^, Dmytro Guzenko^2^, Brian P. Hudson^1^, Yuhe Liang^1^, Robert Lowe^1^, Ezra Peisach^1^, Irina Periskova^1^, Chris Randle^3^, Alexander Rose^3^, Monica Sekharan^1^, Chenghua Shao^1^, Yi-Ping Tao^1^, Yana Valasatava^3^, Maria Voigt^1^, John Westbrook^1^, Jasmine Young^1^, Christine Zardecki^1^, and Marina Zhuravleva^1^

Protein Data Bank Japan:

Genji Kurisu^5^, Haruki Nakamura^5^, Yumiko Kengaku^5^, Hasumi Cho^5^, Junko Sato^5^, Ju Yaen Kim^5^, Yasuyo Ikegawa^5^, Atsushi Nakagawa^5^, Reiko Yamashita^5^, Takahiro Kudou^5^, Gert-Jan Bekker^5^, Hirofumi Suzuki^5^, Takeshi Iwata^5^, Masashi Yokochi^5^, Naohiro Kobayashi^5^, and Toshimichi Fujiwara^5^

Protein Data Bank in Europe:

Sameer Velankar^6^, Gerard J. Kleywegt^6^, Stephen Anyango^6^, David R. Armstrong^6^, John M. Berrisford^6^, Matthew J. Conroy^6^, Jose M. Dana^6^, Mandar Deshpande^6^, Paul Gane^6^, Romana Gáborová^7^, Deepti Gupta^6^, Aleksandras Gutmanas^6^, Jaroslav Koča^7^, Lora Mak^6^, Saqib Mir^6^, Abhik Mukhopadhyay^6^, Nurul Nadzirin^6^, Sreenath Nair^6^, Ardan Patwardhan^8^, Typhaine Paysan-Lafosse^6^, Lukas Pravda^6^, Osman Salih^8^, David Sehnal^6,7^, Mihaly Varadi^6^, and Radka Vařeková^7^

Biological Magnetic Resonance data Bank

John L. Markley^9^, Jeffrey C. Hoch^10^, Pedro R. Romero^9^, Kumaran Baskaran^9^, Dimitri Maziuk^9^, Eldon L. Ulrich^9^, Jonathan R. Wedell^9^, Hongyang Yao^9^, Miron Livny^11^, Yannis E. Ioannidis^12^

^1^ Research Collaboratory for Structural Bioinformatics Protein Data Bank, Institute for Quantitative Biomedicine, Rutgers, The State University of New Jersey, Piscataway, NJ 08854, USA

^2^ Research Collaboratory for Structural Bioinformatics Protein Data Bank, San Diego Supercomputer Center, University of California, San Diego, La Jolla, CA 92093, USA

^3^ Rutgers Cancer Institute of New Jersey, Robert Wood Johnson Medical School, New Brunswick, NJ 08903, USA

^4^ The Scripps Research Institute, La Jolla, CA 92037, USA

^5^ Institute for Protein Research, Osaka University, Suita, Osaka 565-0871, Japan

^6^ Protein Data Bank in Europe, EMBL-EBI, Wellcome Genome Campus, Hinxton, Cambridge CB10 1SD, UK

^7^ CEITEC – Central European Institute of Technology, Masaryk University Brno, Kamencie 5, 625 00 Brno-Bohuice, Czech Republic

^8^ Cellular Structure and 3D Bioimaging, EMBL-EBI, Wellcome Genome Campus, Hinxton, Cambridge CB10 1SD, UK

^9^ Biochemistry Department, University of Wisconsin-Madison, Madison, WI 53706, USA

^10^ Department of Molecular Biology and Biophysics, UConn Health, Farmington, CT 06030, USA

^11^ Department of Computer Sciences, University of Wisconsin-Madison, Madison, WI 53706, USA

^12^ ATHENA Research and Innovation Center, Athens, Greece
